# Fusion membrane proteins derived subunit vaccine candidates effectively protect against *Mycoplasma bovis* challenge in mice

**DOI:** 10.1186/s12917-025-04980-w

**Published:** 2025-10-02

**Authors:** Yong Xuan Wang, Hong Song Cheng, Jun Yue, Qian Hu, Shuai Bo Han, Ying Fen Li, Yu Jie Chen, Er Peng Zhu, Zhen Tao Cheng

**Affiliations:** 1 Department of Veterinary Medicine, College of Animal Science, Guizhou University, Guiyang, 550025 China; 2 Key Laboratory of Animal Diseases and Veterinary Public Health of Guizhou Province, College of Animal Science, Guizhou University, Guiyang, 550025 China; 3 Animal Disease Prevention and Control Center of Guizhou Province, Guiyang, 550001 China

**Keywords:** *Mycoplasma bovis*, Subunit vaccine, Immune efficacy, Membrane proteins

## Abstract

*Mycoplasma bovis* (*M. bovis*) is the causative agent of bovine mycoplasmosis, a disease that can lead to respiratory issues, otitis media, mastitis, and arthritis in cattle and causes huge economic losses to the cattle breeding industry. Although vaccination represents the most effective method for the prevention of *M. bovis*, there is a lack of commercially available subunit vaccines that are effective against this disease. Here, we developed several subunit vaccine candidates using different combinations of membrane proteins M27, M32, M498, and M663 derived from a *M. bovis* strain isolated in Guizhou Province, China. Subsequently, the immune efficacy of the subunit vaccine candidates was evaluated in mice through immunization and challenge experiments. The results showed that the *M. bovis* subunit vaccines constructed from different protein fusions (M27-32, M27-498, M27-663, M27-32–498, M27-32–663, M27-498–663, and M27-32–498-663) were capable of eliciting the secretion of specific antibodies in mice. Furthermore, these candidates also induced robust TNF-α, IFN-γ, IL-4, IL-5, and IL-6 in the serum of mice, suggesting the induction of Th1- and Th2-type immune responses. Microscopically, these *M. bovis* subunit vaccine candidates were also effective in reducing lung tissue damage caused by *M. bovis* infection, suggesting that they provide good protection against challenge with virulent *M. bovis*. Among the tested vaccine candidates, the M27-498–663 and the M27-32–498-663 subunit vaccine candidates demonstrated the most robust immune efficacy, laying the foundation for the effective control of *M. bovis*.

**Supplementary Information:**

The online version contains supplementary material available at 10.1186/s12917-025-04980-w.

## Background

*Mycoplasma bovis* (*M. bovis*) was first isolated from sick cows with mastitis in the United States in 1961 [1]. In China, the first isolation of *M. bovis* from the lungs of calves with pneumonia was reported in 2008 [2]. Infection with *M. bovis* can manifest in a number of ways, including mastitis, pneumonia, arthritis, keratoconjunctivitis, otitis media, and genital disorders that may result in infertility and abortion [3]. Therefore, *M. bovis* represents a significant threat to cattle health and productivity, with a global prevalence that has resulted in considerable economic losses for the cattle industry [4].

The membrane proteins on the surface of the Mycoplasma cell membrane play a crucial role in the invasion process, due to the absence of cell walls. The latest research indicates that *M. bovis* primarily disrupts the function of membrane surface receptors on host cells by secreting inflammatory chemicals and activating inflammatory signaling pathways through membrane protein cilia. Such an interaction may result in damage to the host cell membrane and, in some cases, may even lead to apoptosis [5]. A genomic cluster of variable surface lipoproteins (Vsp) genes (*VspA*, *VspB*, *VspC*, *VspF*, *VspO*, *and VspL*) mediated high-frequency phenotypic switching of surface lipoprotein antigens in the bovine pathogen *M. bovis*. This alteration serves to enhance the ability of the organism to colonize, attach to, and evade host immune defenses. These membrane proteins are highly immunogenic and induce mucosal immunity [6]. Given the absence of a cell wall, the antibiotic arsenal available for the treatment of *M. bovis* infections is constrained [7]. The increasing resistance of *M. bovis* to multiple antibiotics is a growing concern, as it places a financial burden on farmers due to the elevated cost of treatments. Consequently, vaccination represents a valuable alternative for the prevention and control of *M. bovis* infection [8]. To achieve effective control of *M. bovis* infection, structurally conserved membrane proteins with multiple antigenic epitopes can be utilized for diagnostic characterization and vaccine development [6]. However, there is a lack of commercially available subunit vaccines that are effective against this disease currently. In mycoplasmas, the integral and membrane-associated proteins are exposed to the external environment and play an important role in the survival and pathogenesis of the agent. Mycoplasmas possess a number of several lipid-associated membrane proteins (LPPs) that are able to modulate immune responses [7]. In light of the research trend concerning membrane proteins of *M. bovis* and the previous research outcomes on *M. bovis* within our research group, this experiment was carried out with the four well-conserved *M. bovis*-associated membrane proteins (M27, M32, M498, and M663) that have numerous antigenic epitopes related to adhesion screened previously [8]. The objective of this study was to develop multi-protein subunit vaccine candidates using different combinations of four *M. bovis* membrane proteins (M27, M32, M498, and M663) with conserved and antigenic properties. The efficacy of the vaccine candidates was assessed through immunization and challenge tests in mice. These results will serve as references for further research into the development of novel *M. bovis* vaccines.

## Materials and methods

### Strains, cells, plasmid, and serum

The *M. bovis* Guizhou strain used in this study was isolated and preserved in our laboratory (Yuan et al. 2017). The prokaryotic expression vector pCold Ⅰ and recombinant expression vectors pCold-*M27*, pCold-*M32*, pCold-*M498*, and pCold-*M663* were stocked in our laboratory. The *Escherichia coli* DH5*α* and BL21 (DE3) strains were purchased from Tiangen Biochemical Technology Co., Ltd, (Beijing, China). The premium horse serum (16,050,122) was purchased from Gibco (USA).

### Expression, purification, and characterization of the recombinant fusion proteins

Specific primers (GenBank: NC_014760.1) were designed to amplify the *M27*, *M32*, *M498*, and *M663* genes using the pCold-*M27*, pCold-*M32*, pCold-*M498*, and pCold-*M663* plasmid as templates, respectively. Next, the fusion genes *M27-32* (1 020 bp), *M27-498* (1 167 bp), *M27-663* (1 248 bp), *M32-498* (870 bp), *M32-663* (951 bp), *M498-663* (1 101 bp), *M27-32–498* (1 526 bp), *M27-32–663* (1 620 bp), *M27-498–663* (1 770 bp), *M32-498–663* (1 473 bp), and *M27-32–498-663* (2 160 bp) were amplified using splicing by overlap extension-polymerase chain reaction (SOE-PCR). Primer information is listed in Table 1. These genes were then subcloned into the pCold I vector using restriction enzymes *Hin*d Ⅲ and *Xba* I (TaKaRa, Japan), respectively. Based on the nucleotide sequence of FAdV-4 ON1 strain (GenBank: GU188428). The obtained constructs (pCold-M27-32, pCold-M27-498, pCold-M27-663, pCold-M32-498, pCold-M32-663, pCold-M498-663, pCold-M27-32–498, pCold-M27-32–663, pCold-M27-498–663, pCold-M32-498–663, and pCold-M27-32–498-663) were identified and transformed into *E. coli* BL21 (DE3) and then cultured in a shaker at 170 r/min and 37 ℃. Once *D*_600nm_ reached 0.6–0.8, the culture was induced with 1 mmol/L isopropyl-β-D-thiogalactopyranoside (IPTG) for 3 h at 170 r/min and 16 ℃ for the expression of recombinant proteins.

**Table 1:** Primers used for the PCR reactions

Primer name	Primer sequence (5’−3’)
M27-F1	GG *GGTACC* ATGAAAGGTAAAAAGCCAGAAAGT
M27-R1	GCTTCCGCCACCGCCGCTTCCACCGCCACCATTTATTTTCTCATTATTAAT
M32-F1	GG *GGTACC* ATGATAAAAAAATCTAATAACAAT
M32-R1	GCTTCCGCCACCGCCGCTTCCACCGCCACCGTGAACAATTAACTTGGAAT
M32-R2	GGTGGCGGTGGAAGCGGCGGTGGCGGAAGCATGATAAAAAAATCTAATAACAAT
M32-R3	CG *GGATCC* TTAGTGAACAATTAACTTGGAAT
M498-F1	GG *GGTACC* ATGAAAAAGCATAAATTAGAAA
M498-R1	GGTGGCGGTGGAAGCGGCGGTGGCGGAAGCAAAAAGCATAAATTAGAAA
M498-R2	GGGGGGGGAGGTAGCGGAGGCGGAGGTAGCAAAAAGCATAAATTAGAAA
M498-R3	CG *GGATCC* TTATTCACCTCTGAATAATGCGCT
M663-F1	GG *GGTACC* ATGAAAGGTAAAAAGCCAGAAAGT
M663-R1	GGGGGGGGAGGTAGCGGAGGCGGAGGTAGCTTTCTTTTGCGTACTTTGA
M663-R2	GGTGGCGGTGGAAGCGGCGGTGGCGGAAGCAAAGGTAAAAAGCCAGAAAGT
M663-R3	CG *GGATCC* TTATTTCTTTTGCGTACTTTGA

*Note*: “___” indicates the linker; The Italic letter indicates restriction enzyme cleavage site

The samples were prepared and then analyzed using sodium dodecyl sulfate–polyacrylamide gel electrophoresis (SDS-PAGE). For the sake of convenience, the recombinant fusion membrane proteins were named as fusion proteins M27-32, M27-498, M27-663, M32-498, M32-663, M498-663, M27-32–498, M27-32–663, M27-498–663, M32-498–663, and M27-32–498-663, respectively. These proteins were purified using the His-Tag protein purification kit (OMEGA, USA), and the purified products were subjected to SDS-PAGE analysis and Western blotting using a Rabbit-derived positive serum against *M. bovis* and a goat anti-rabbit immunoglobulin G (IgG)-horseradish peroxidase (HRP) (Biosynthesis Biotechnology Co., Ltd, Beijing, China).

### Immunization and challenge experiment

**Fig. 1:**
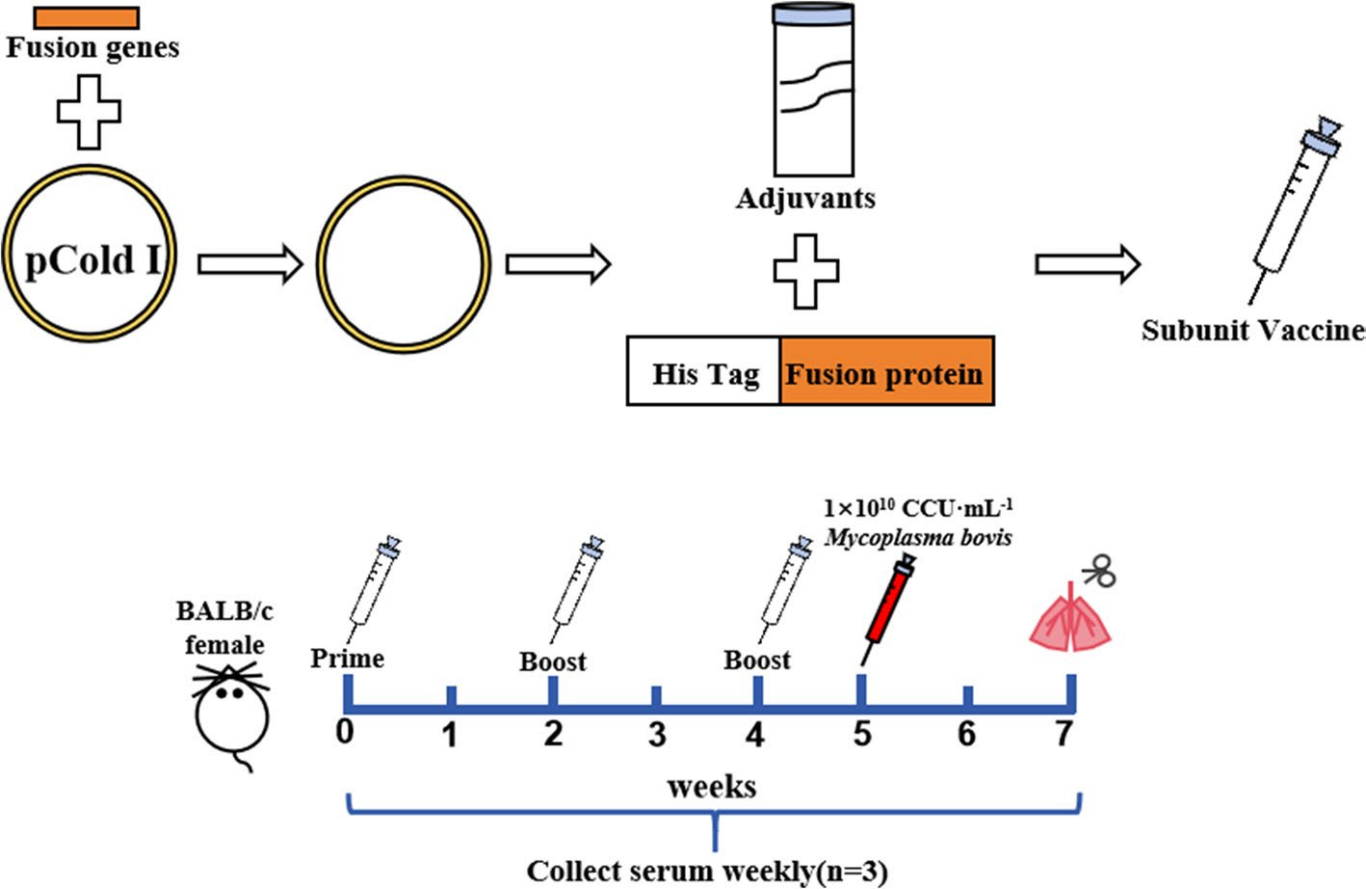
Schematic diagram of the immunization and challenge experiment in mice. 420 mice were randomly divided into 12 groups (*n* = 35 per group) and immunized intramuscularly thrice with PBS or different subunit vaccine candidates. The immunizations were administered at two-week intervals. One week after the final immunization, mice (*n* = 4) were randomly selected from each group and then challenged by i.m. in the leg with a minimum infectious dose (1 × 10^10^ CCU·mL^−1^) of *M.* bovis. The lungs were collected from mice euthanized two weeks after the challenge for histopathological analysis

Protein concentration was determined using the BCA protein quantification kit. The subunit vaccine candidates were prepared by combining the expressed fusion proteins with Freund's adjuvant (1:1) for emulsification. To assess the immune effects of different *M. bovis* subunit vaccine candidates, experimental SPF female mice (*n* = 420) aged 4 weeks of age were randomly divided into 12 groups (*n* = 35). The vaccine candidates were administered via hypodermic injection (i.h.) into the neck and back. Likewise, subsequent booster immunizations were conducted at two-week intervals for a total of three injections. The detailed immunization procedures are listed in Table 2. The mice in each group were raised separately. Serum was collected separately from each group of mice (n = 3) per week. Serum was collected eight times from the pre-immunization period to the seventh week after immunization. One week after the final immunization, four mice were randomly selected from each group and then challenged via intramuscular injection in the leg with a minimum infectious dose (1 × 10^10^ CCU·mL^−1^) of *M. bovis*. The detailed immunization procedures in Table 2. The clinical symptoms of mice were recorded within 7 days. The presence of abnormal signs was judged to be morbid. The morbidity rate of mice in each experimental group was counted. The lungs were collected from mice euthanized two weeks after the challenge for histopathological analysis. Briefly, the lungs were fixed with 4% paraformaldehyde fixative and subsequently underwent a graded dehydration process with ethanol. The tissue block was made transparent using xylene and paraffin embedding, after which it was sliced, patched, and baked. The sections were dewaxed and stained with hematoxylin–eosin (HE). Finally, the slices were sealed with neutral gum and then observed under the microscope to examine the pathological changes in the lung tissue. The experimental design is shown in Fig. 1.

**Table 2:** The detailed immunization procedures in this study

Groups	Dosage per mice	Numbers of mice	Immunization	
			Times	Route
PBS	100 μL	35	3	i.h
M27-32 subunit vaccine	100 μg	35	3	i.h
M27-498 subunit vaccine	100 μg	35	3	i.h
M27-663 subunit vaccine	100 μg	35	3	i.h
M32-498 subunit vaccine	100 μg	35	3	i.h
M32-663 subunit vaccine	100 μg	35	3	i.h
M498-663 subunit vaccine	100 μg	35	3	i.h
M27-32–498 subunit vaccine	100 μg	35	3	i.h
M27-32–663 subunit vaccine	100 μg	35	3	i.h
M27-498–663 subunit vaccine	100 μg	35	3	i.h
M32-498–663 subunit vaccine	100 μg	35	3	i.h
M27-32–498-663 subunit vaccine	100 μg	35	3	i.h

### Detection of fusion protein-specific antibody and cytokine secretion by Enzyme-Linked Immunosorbent Assay (ELISA)

In this study, an indirect ELISA method was established for the detection of fusion protein specific antibody. Briefly, the purified fusion protein M27-32–498-663 was diluted as coated antigen and incubated in a 96-well plate overnight at 4 °C. After a one-hour blockage at 37 °C with 5% skim milk and three washes with Phosphate-Buffered Saline with Tween 20 (PBST), diluted serum samples were added to the coating plates and incubated for one hour at 37 °C. Blank control wells were set up. After three washes, the goat anti-mouse IgG HRP conjugate was added to each well, and incubation was performed for 1 h at 37 °C. After three washes, 50 μL of the chromogenic solution was added to each well and incubated in a light-protected environment at room temperature for 15 min. The reaction was terminated by the addition of 50 µL of terminator solution to each well, and the absorbance was recorded at 450 nm. Samples with an OD450 nm value less than 0.268 are considered negative, while those with an OD450 nm value greater than 0.268 are considered positive.

The secretion of Th1-type cytokines TNF-α (JYM0218Mo), IFN-γ (JYM0540Mo), and Th2-type cytokines IL-4 (JYM0011Mo), IL-5 (JYM0191Mo), and IL-6 (JYM0012Mo) (Wuhan Colorful-Gene biological technology Co., Ltd, China) in mouse serum was respectively measured according to the instructions for the mouse cytokine ELISA kits. Finally, the absorbance was recorded at 450 nm. A standard curve was plotted using the concentration of the standard sample on the abscissa and the OD450nm value on the ordinate. A regression equation for the standard curve was obtained. According to the OD450nm of the sample, the actual concentration of cytokines in each sample can be measured.

### Statistical analysis

The results are shown as mean ± standard deviations (SD) of at least three independent experiments and were analyzed by the one-way ANOVA tests using the Prism 7.0 software. *P*-value < 0.05 denotes the statistical significance of the data.

## Result

### Expression and identification of the fusion proteins of M. bovis

**Fig. 2:**
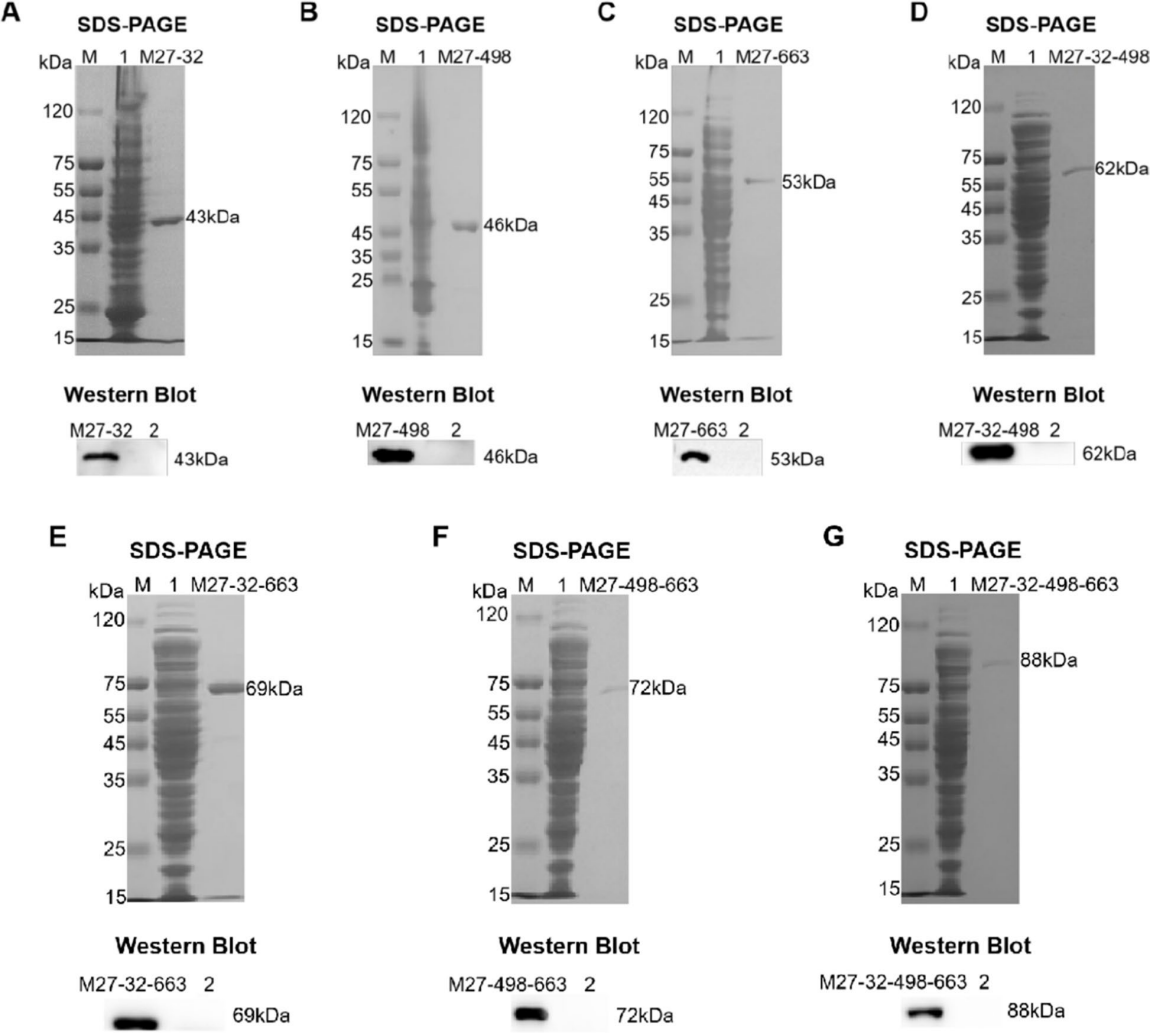
SDS-PAGE and Western blot analysis of the purified fusion proteins. M: Protein marker. Lane 1: Unpurified fusion proteins. Lane 2: pColdⅠ empty plasmid bacterial culture. **A** SDS-PAGE and Western blot analysis of the purified fusion protein M27-32 (43 kDa). **B** SDS-PAGE and Western blot analysis of the purified fusion protein M27-498 (46 kDa). **C** SDS-PAGE and Western blot analysis of the purified fusion protein M27-663 (53 kDa). **D** SDS-PAGE and Western blot analysis of the purified fusion protein M27-32–498 (62 kDa). **E** SDS-PAGE and Western blot analysis of the purified fusion protein M27-32–663 (69 kDa). **F** SDS-PAGE and Western blot analysis of the purified fusion protein M27-498–663 (72 kDa). **G** SDS-PAGE and Western blot analysis of the purified fusion protein M27-32–498-663 (88 kDa)

Recombinant plasmids were transformed into *E.coli* BL21 (DE3), and the recombinant proteins were expressed through IPTG induction. Subsequently, the expression of each fusion protein was detected by SDS-PAGE and Western blot analysis. SDS-PAGE analysis showed that a total of seven fusion proteins were obtained in this study. The purified fusion proteins were subjected to Western blot analysis, which exhibited specific bands of target proteins at the expected positions (Fig. 2A-G). These results suggest that the purified fusion proteins possess good reactogenicity, laying foundations for the development of the *M. bovis* fusion membrane protein subunit vaccine candidates.

### M. bovis membrane protein subunit vaccine candidates induce high-level specific antibodies in mice sera

**Fig. 3:**
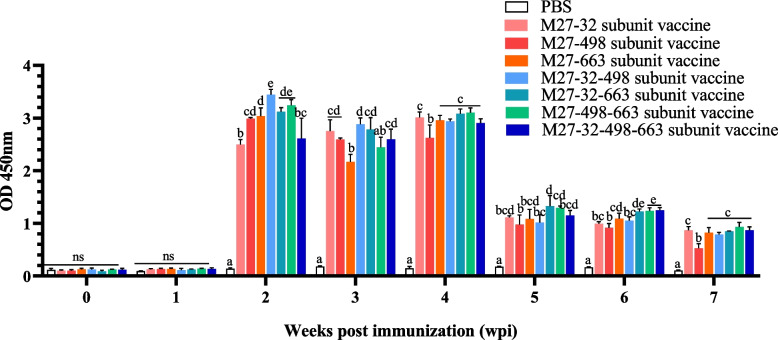
Dynamics of fusion protein specific antibodies in the serum of immunized mice (*n* = 35). Mice were immunized three times at 2-week intervals with PBS or subunit vaccines (M27-32, M27-498, M27-663, M27-32–498, M27-32–663, M27-498–663, and M27-32–498-663). Serum samples were collected 7 days after each immunization, and antigen-specific antibodies were measured by indirect ELISA. All samples were analyzed in triplicate. Comparisons were performed by one-way ANOVA. Statistical significance was denoted by different lowercase letters (*P* < 0.05), while Ns and same lowercase letters indicated no significant difference

To evaluate humoral immune response elicited by the different *M. bovis* subunit vaccines, the levels of specific antibodies in the sera of the immunized mice were determined by ELISA. Compared to the PBS group (control), all *M. bovis* subunit vaccine candidates induced a statistically significant increase (*P* < 0.05) in specific antibody levels, reaching a peak at 2 weeks post-immunization (wpi). These data indicate that all fusion protein subunit vaccines induced robust humoral immune responses (Fig. 3).

### M. bovis membrane protein subunit vaccine candidates induce robust Th1- and Th2-type responses in mice sera

**Fig. 4:**
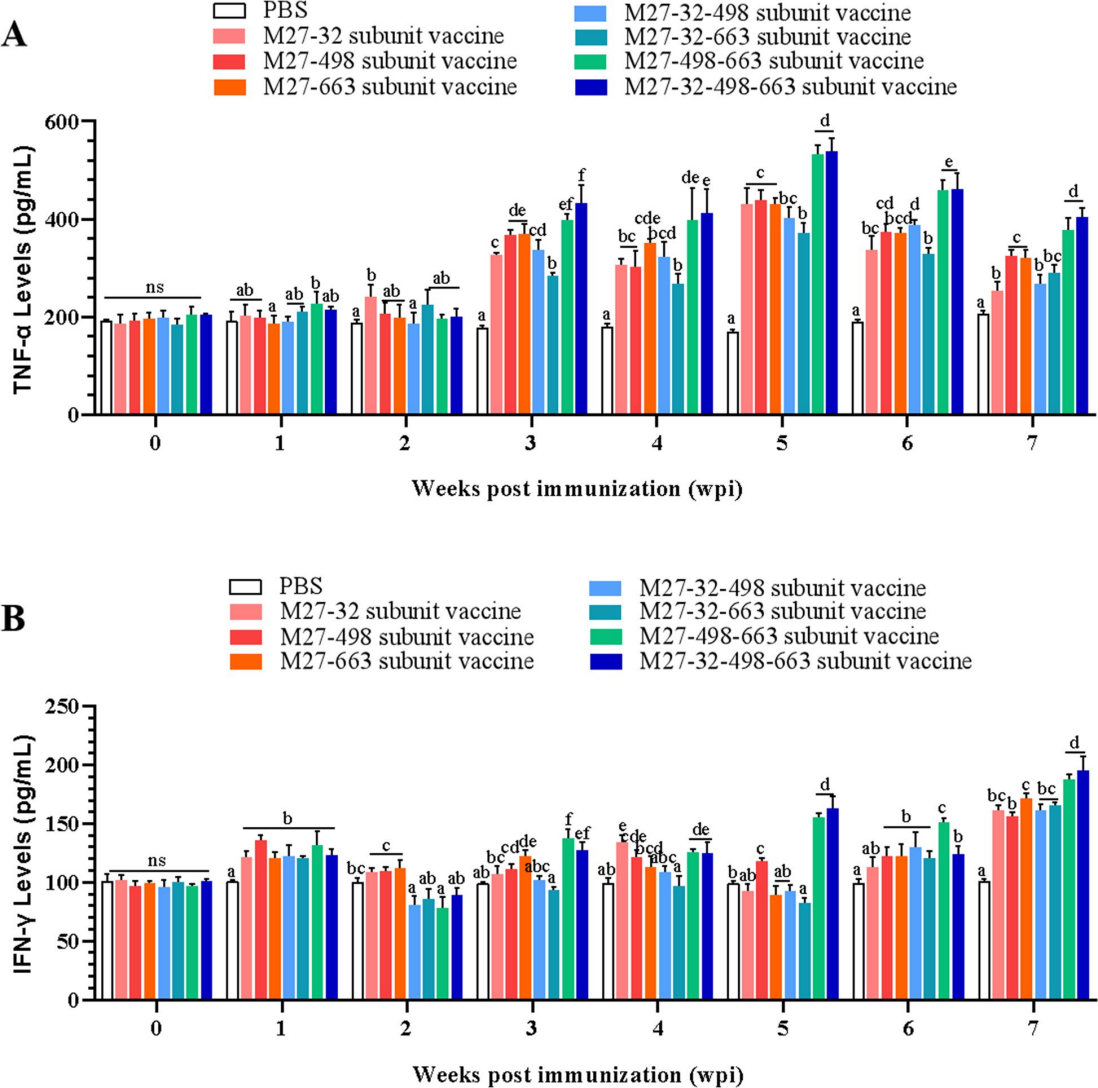
Vaccination elevates the production of Th1-type cytokines in mice serum. **A** Vaccination induces the dynamic changes of TNF-α in mice serum. **B** Vaccination induces the dynamic changes of IFN-γ in mice serum. Mice were immunized three times at 2-week intervals with PBS or subunit vaccines (M27-32, M27-498, M27-663, M27-32–498, M27-32–663, M27-498–663, and M27-32–498-663). Serum samples were collected 7 days after each immunization, and Th1-type cytokines levels were measured by indirect ELISA. Comparisons were performed by one-way ANOVA. Statistical significance was denoted by different lowercase letters (*P* < 0.05), while Ns and same lowercase letters indicated no significant difference

To investigate the vaccine-induced cellular immune responses, secretion levels of Th1-type cytokines (IFN-γ and TNF-α) in the sera of the immunized mice were determined by ELISA. Compared with the PBS group (control), all fusion protein subunit vaccine candidates stimulated robust expression of IFN-γ and TNF-α in mice. The highest levels of TNF-α secretion were observed in the immunized group at 5 wpi, while the peak levels of IFN-γ secretion were reached at 7 wpi in all experimental groups. These results indicate that the fusion protein vaccine candidates were all efficacious in stimulating Th1-type cellular immune responses in mice. Furthermore, a comparative analysis of the amount and trend of TNF-α and IFN-γ secretion throughout the trial period revealed that the vaccine candidates based on the M27-498–663, and M27-32–498-663 fusion proteins possess better immunization effects than other groups(*P* < 0.05) (Fig. 4 A and B).

**Fig. 5:**
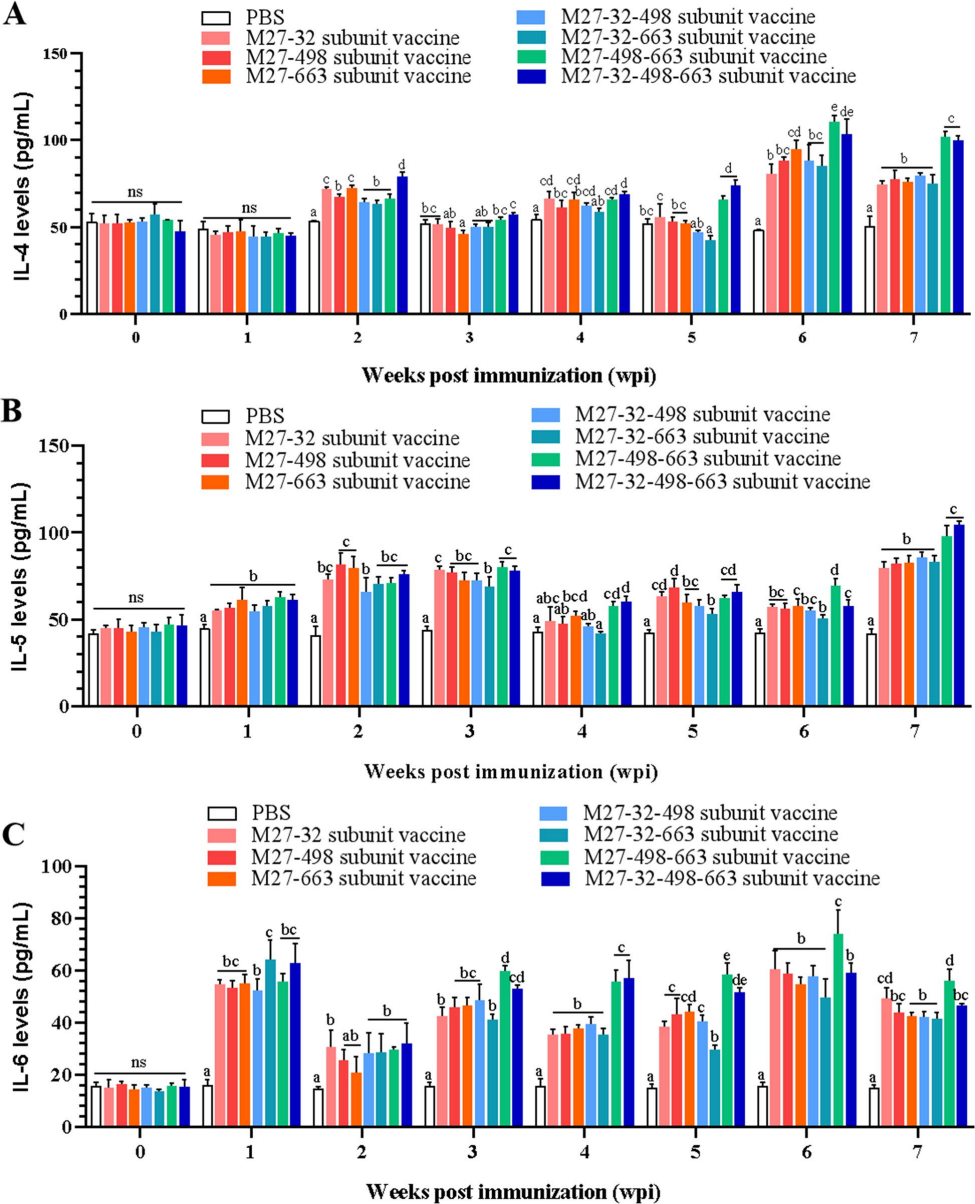
Vaccination induces the production of Th2-type cytokines in mice serum. **A** Vaccination induces the levels of IL-4 in mice serum. **B** Vaccination induces the levels of IL-5 in mice serum. **C** Vaccination induces the levels of IL-6 in mice serum. Mice were immunized three times at 2-week intervals with PBS or subunit vaccines (M27-32, M27-498, M27-663, M27-32–498, M27-32–663, M27-498–663, and M27-32–498-663). Serum samples were collected 7 days after each immunization, and Th2-type cytokines levels were measured by indirect ELISA. Comparisons were performed by one-way ANOVA. Statistical significance was denoted by different lowercase letters (*P* < 0.05), while Ns and same lowercase letters indicated no significant difference

To further evaluate the humoral immune responses induced by the prepared vaccine candidates, the levels of Th2-type cytokines (IL-4, IL-5, and IL-6) in the serum of immunized mice were measured by ELISA. Compared with the PBS group, all vaccine candidates enhanced ability to stimulate IL-4 secretion in mice, with the M27-498–663 subunit vaccine having the best effect on IL-4 secretion in mice (Fig. 5A). Furthermore, all vaccine immunization groups increased secretion IL-5 in comparison with the PBS group, among which the M27-32–498-663 subunit vaccine group exhibited more effective stimulation of IL-5 secretion in mice (Fig. 5B). Additionally, all vaccine groups stimulated mice to produce high levels of IL-6, which remained high throughout the test period. Among them, the M27-498–663 and the M27-32–498-663 subunit vaccines were more effective in stimulating IL-6 secretion in mice (Fig. 5C). Collectively, the results displayed above indicate tests showed that all the prepared *M. bovis* subunit vaccines were able to stimulate Th1 and Th2-type cellular immune responses in mice.

### M. bovis membrane protein subunit vaccine candidates attenuate the damage to the lungs induced by M. bovis challenge in mice

**Fig. 6:**
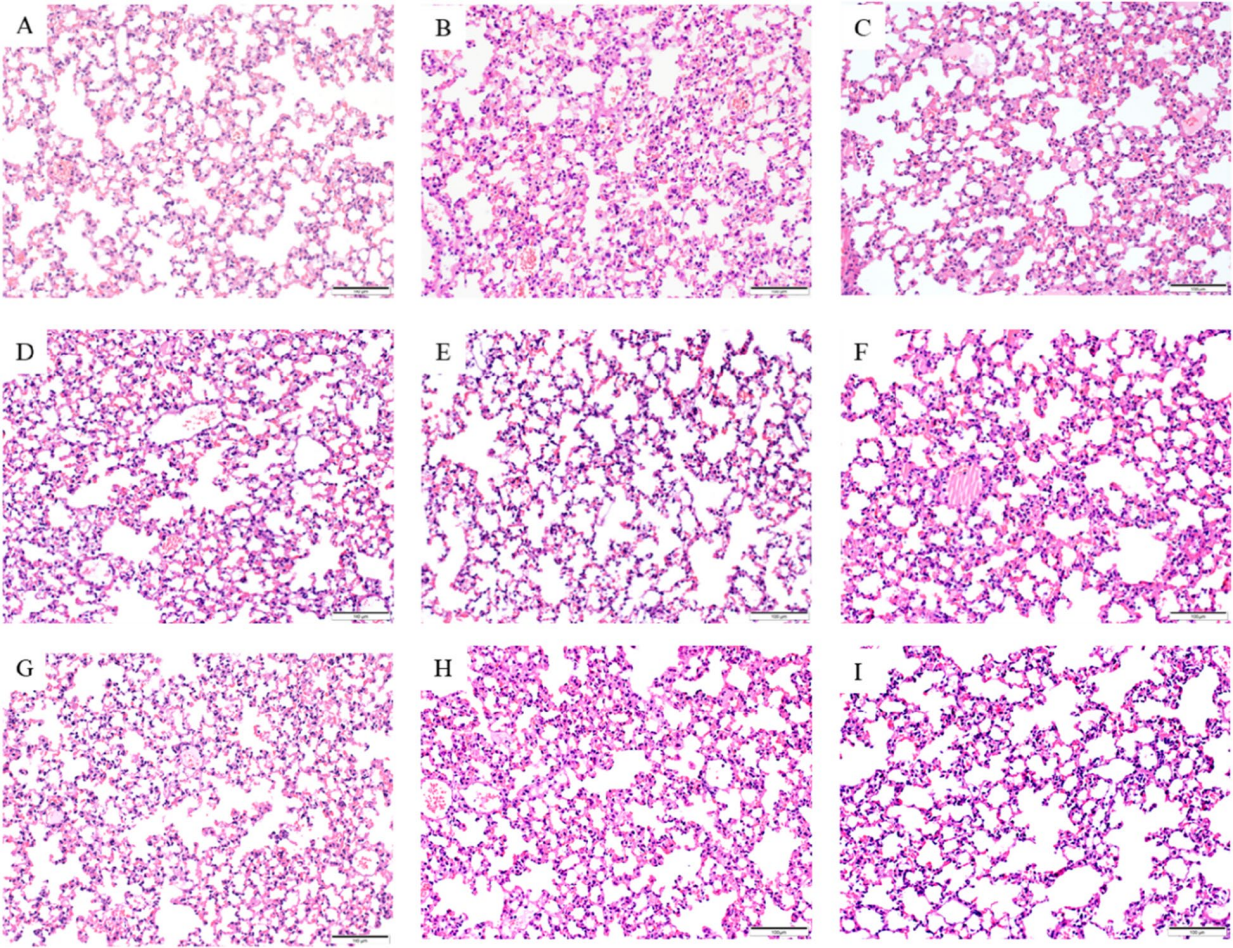
M27-32–498-663 subunit vaccine effectively ameliorates *M. bovis*-induced lung injury in mice. **A** Lung sections of normal mice. **B** Lung sections of *M. bovis* infected mice immunized with PBS. **C** After *M. bovis* infection, M27-32–498 subunit vaccine immunized mice showed slight alterations in alveolar structure, thickening of alveolar walls, and inflammatory cell infiltration. **D** Mice immunized with the M27-32 subunit vaccine infected with *M. bovis* showed slight changes in alveolar structure, thickening of the alveolar wall, and partial infiltration of inflammatory cells. **E**/**F**/**G** The subunit vaccine candidates M27-498, M27-663, and M27-32–663*-*immunized mice after *M. bovis* infection demonstrated subtle architectural changes of alveoli featuring focal alveolar wall thickening and minimal inflammatory cell infiltration in pulmonary tissue. **H ***M. bovis* infection of mice immunized with the M27-498–663 subunit vaccine resulted in a more intact alveolar structure with a slight thickening of the alveolar wall and a small amount of inflammatory cell infiltration. **I** Mice immunized with the M27-32–498-663 subunit vaccine followed by challenge with *M. bovis* showed a more intact alveolar structure, with no significant thickening of the alveolar wall and merely a small amount of inflammatory cell infiltration. Bar: 100 μm

To assess the immunoprotective efficacy of the subunit vaccine candidates, four mice from each test group were infected with the minimum infectious dose (1 × 10^10^ CCU·mL^−1^) of *M. bovis* via intramuscular injection one week following the final immunization. The lung tissues of mice in each group were collected two weeks after the challenge, and tissue sections were prepared according to the conventional method. The pathological alterations in each group were then observed using a microscope after HE staining. The normal mouse alveoli are structurally intact, and no evidence of inflammation is present (Fig. 6A). In mice that had been immunized with PBS and subsequently infected with *M. bovis*, the alveoli formed fused foci of agglomerates, with degeneration or loss of alveolar structure, fracture of the alveolar wall, and infiltration of a large number of inflammatory cells (Fig. 6B). After *M. bovis* infection, M27-32–498 subunit vaccine immunized mice exhibited subtle alterations in alveolar structure, such as thickening of alveolar walls, and infiltration of inflammatory cells (Fig. 6C). Mice immunized with the M27-32 subunit vaccine infected with *M. bovis* showed slight alterations in alveolar structure, including thickening of the alveolar wall and partial infiltration of inflammatory cells (Fig. 6D). The subunit vaccine candidates M27-498, M27-663, and M27-32–663*-*immunized mice after *M. bovis* infection demonstrated subtle architectural changes of alveoli featuring focal alveolar wall thickening and minimal inflammatory cell infiltration in pulmonary tissue (Fig. 6E/F/G). *M. bovis* infection of mice immunized with the M27-498–663 subunit vaccine resulted in a more intact alveolar structure with a slight thickening of the alveolar wall and a small amount of inflammatory cell infiltration (Fig. 6H). Of note, mice immunized with the M27-32–498-663 subunit vaccine candidate followed by challenge with *M. bovis* showed a more intact alveolar structure, with no significant thickening of the alveolar wall and merely a minimal amount of inflammatory cell infiltration (Fig. 6I). Taken together, the subunit vaccine-immunized mice exhibited a relatively intact alveolar structure, a relatively lesser degree of alveolar wall thickening, a reduced number of inflammatory cells infiltrating the lungs, and a diminished extent of inflammatory manifestations in response to *M. bovis* infection, when compared to the control group. These results indicate that immunization with the vaccine candidates mitigates lung tissue damage caused by *M. bovis* infection in mice.

## Discussion

*M. bovis* is highly contagious. And susceptibility of *M. bovis* to various antimicrobial classes targeting protein synthesis and DNA synthesis is decreased [9]. The main focus in the cattle industry is on the prevention of the disease, so the development of novel *M. bovis* vaccines is critical [10]. There are two commercially available *M. bovis* vaccines (Mycomune@R and Pulmo-GuardTMMpB), but they are not very effective [11]. Musa Mulongo et al. prepared two traditional subunit vaccines against *M. bovis* (*M. bovis* total extracts and membrane fractions). However, the experimental results show that the vaccines did not offer protection against challenge with an infective dose of *M. bovis* [12]. In the present study, we developed a series of genetically engineered subunit vaccine candidates using different combinations of four conserved membrane proteins of *M. bovis* that induced specific antibodies and Th1/Th2-type responses in mice sera, and ameliorated M. bovis-induced lung tissue damage in mice. While murine models provide proof-of-concept, protective efficacy in cattle may differ due to species-specific immune variations. These results support further evaluation of these antigens in bovine vaccine development.

To enhance the immune responses in subunit vaccines, we elected to augment the number of antigenic species. Four membrane protein genes were combined in varying quantities and expressed as fusion membrane proteins using genetic engineering techniques to obtain multiple fusion proteins. The results showed that we successfully expressed a series of fusion proteins, including M27-32, M27-498, M27-663, M27-32–498, M27-32–663, M27-498–663, and M27-32–498-663, respectively.

Mycoplasma can infect poultry, dogs, mice, cattle and other animals [13]. There are many reports of mycoplasmas in hosts that are not perceived as their normal habitat. Sometimes these “crossings” may have a pathological impact particularly where there may be predisposing conditions [14]. Anderson et al. also showed that Mycoplasma bovis exhibited pathogenicity in mice similar to that of cattle [15]. Therefore, BALB/c mice were chosen as test animals for this experiment. Immunization of mice with subunit vaccine prepared from fusion proteins to explore the immunoprotective effect of fusion proteins. Recognizing that immune responses may differ between murine and bovine systems.

Serum-specific antibody levels are a good indicator of humoral response Zhang Y et al. demonstrated that *M. bovis* can be killed directly by complement and that antibody-dependent complement-mediated killing is more effective than that by complement alone [16]. In this study, we found that all vaccine candidates elicited significantly higher specific antibodies compared to the PBS control group (*P* < 0.05). This suggested that the respective membrane proteins promoted the production of antigen-specific neutralizing antibodies and stimulated protective immunity. The overall secretion trend showed a peak 14 days after the first immunization, followed by a decline. There was a rebound 14 d after the second immunization, and no significant increase was seen after the third immunization, but it was relatively stable. The M27-498–663 subunit vaccine antibody was secreted at the highest level.

Cytokines, primarily produced by macrophages, antigen presenting cells and lymphocytes, activate the immune system in response to infection, particularly infection occurring at mucosal surfaces. TNF-α and IFN-γ expression it should be associated with increased clinical course and severity of lung lesions during *M. Bovis* pneumonia [16]. *M. bovis* induces mixed Th1-Th2 cytokine responses in *M. Bovis*-associated lung lesions. Rodríguez et al. demonstrated consistent upregulation of TNF-α, IL-4, and IFN-γ expression during *M. bovis*-associated pneumonic lesions. These cytokines can participate in the immune and inflammatory responses during the pulmonary defense mechanisms against *M. bovis* infection [17]. Vanden Bush TJ et al. suggest that experimental lung infection of cattle with *M. bovis* results in a Th2-skewed immune response [18]. In the present study, all the *M. bovis* subunit vaccines prepared stimulated the body to produce Th1 (TNF-α and IFN-γ) and Th2 (IL-4, IL-5, IL-6) type cellular immune responses. The inflammatory response in the lungs of mice in each vaccine-immunized attack group was attenuated compared with the control attack group. This suggests that each *M. bovis* membrane protein vaccine immunization group increased the resistance of mouse lungs to *M. bovis* infection. This may be related to an increase in antigens, or some combination of specific antigens. In addition, the pathological examination revealed that all vaccine groups exhibited less severe lung tissue damage compared to the control group, with the M27-32–498-663 subunit vaccine group showing the most preserved pulmonary architecture. These findings suggest that immunization with these membrane protein formulations was associated with attenuated inflammatory responses to M. bovis challenge in this murine model. While histological assessments were qualitative, future studies would benefit from quantitative metrics (e.g., alveolar wall thickness or standardized inflammatory scoring) to enhance objectivity, though sample constraints precluded this analysis herein.

While these results are promising, several considerations should be noted: (1) the protective efficacy was evaluated only in a murine model, (2) the immunological correlates of protection remain to be fully elucidated, and (3) the optimal adjuvant formulation requires further optimization. Subsequent studies will focus on refining the vaccine production process and investigating alternative adjuvant combinations in expanded mouse trials before progressing to bovine studies.

## Conclusion

Results indicate that all *M. bovis* membrane protein subunit vaccine candidates constructed in this study induce significant specific antibodies and Th1 and Th2-type cytokine responses in mice. Of these, M27-498–663 and M27-32–498-66 subunit vaccine candidates demonstrate better potential as effective *M. bovis* subunit vaccine candidates in the future. While these murine data provide proof-of-concept, further studies are needed to: (1) optimize adjuvant formulations, (2) evaluate efficacy in the natural bovine host, and (3) assess long-term protection.

## Supplementary Information

Supplementary Material 1.

Supplementary Material 2.

## Data Availability

Data is provided within the manuscript or supplementary information files.
