# Supplementary figures and images for: Fusion membrane proteins derived subunit vaccine candidates effectively protect against Mycoplasma bovis challenge in mice
Source: BMC Vet Res. 2025 Oct 2;21:559. doi: 10.1186/s12917-025-04980-w (PMC12492813; doi:10.1186/s12917-025-04980-w)

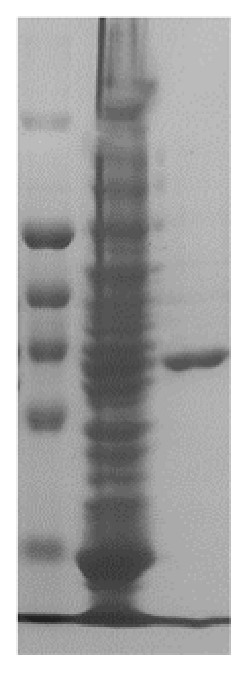

Supplement: Supplementary file 1 — Supplementary Material 1. [file 12917_2025_4980_MOESM1_ESM.zip › Uncropped_gels_blots_Figure.2/SDS-PAGE/Fig.2 A Unlabeled.jpg]

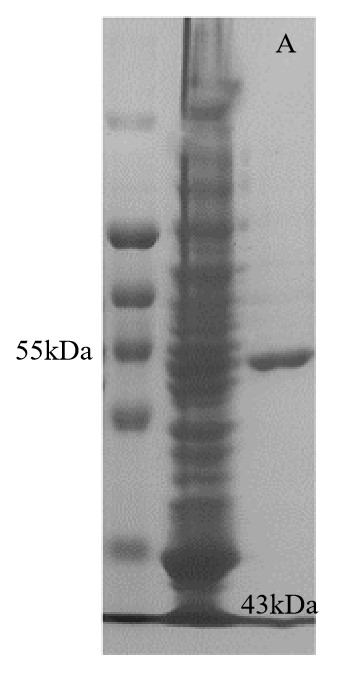

Supplement: Supplementary file 1 — Supplementary Material 1. [file 12917_2025_4980_MOESM1_ESM.zip › Uncropped_gels_blots_Figure.2/SDS-PAGE/Fig.2 A.jpg]

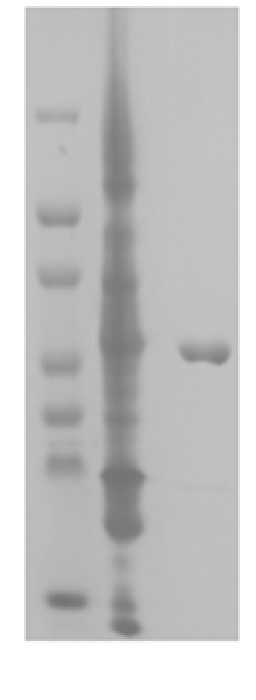

Supplement: Supplementary file 1 — Supplementary Material 1. [file 12917_2025_4980_MOESM1_ESM.zip › Uncropped_gels_blots_Figure.2/SDS-PAGE/Fig.2 B Unlabeled.jpg]

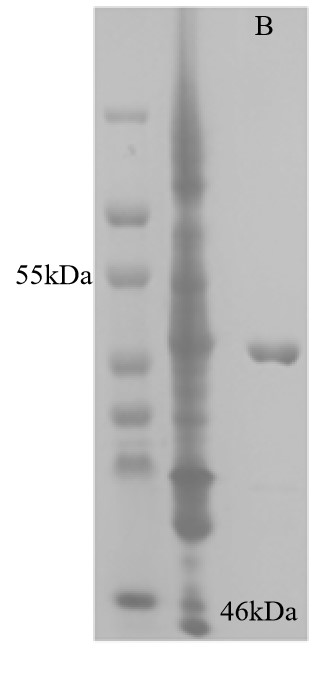

Supplement: Supplementary file 1 — Supplementary Material 1. [file 12917_2025_4980_MOESM1_ESM.zip › Uncropped_gels_blots_Figure.2/SDS-PAGE/Fig.2 B.jpg]

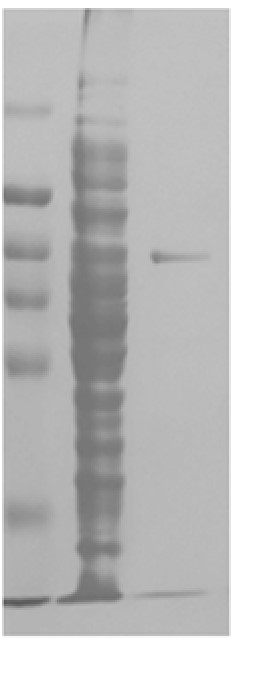

Supplement: Supplementary file 1 — Supplementary Material 1. [file 12917_2025_4980_MOESM1_ESM.zip › Uncropped_gels_blots_Figure.2/SDS-PAGE/Fig.2 C Unlabeled.jpg]

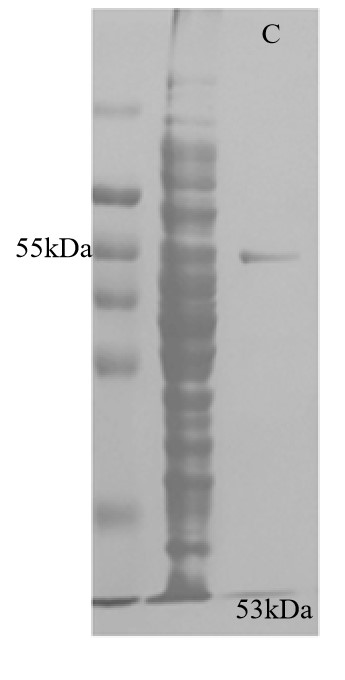

Supplement: Supplementary file 1 — Supplementary Material 1. [file 12917_2025_4980_MOESM1_ESM.zip › Uncropped_gels_blots_Figure.2/SDS-PAGE/Fig.2 C.jpg]

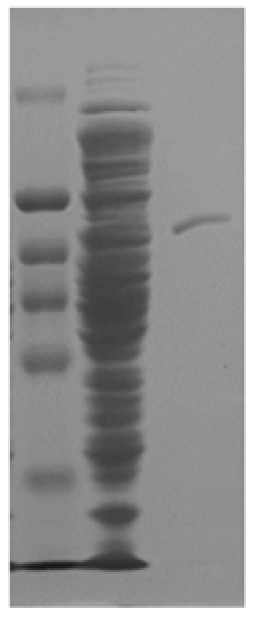

Supplement: Supplementary file 1 — Supplementary Material 1. [file 12917_2025_4980_MOESM1_ESM.zip › Uncropped_gels_blots_Figure.2/SDS-PAGE/Fig.2 D Unlabeled.jpg]

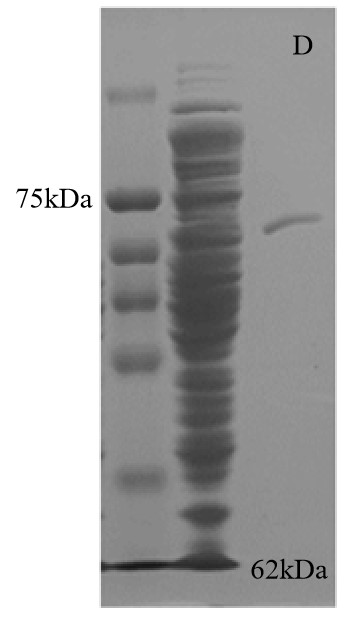

Supplement: Supplementary file 1 — Supplementary Material 1. [file 12917_2025_4980_MOESM1_ESM.zip › Uncropped_gels_blots_Figure.2/SDS-PAGE/Fig.2 D.jpg]

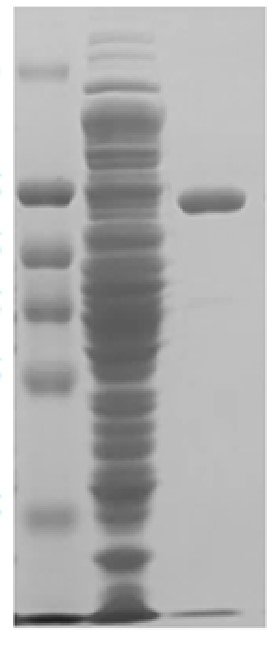

Supplement: Supplementary file 1 — Supplementary Material 1. [file 12917_2025_4980_MOESM1_ESM.zip › Uncropped_gels_blots_Figure.2/SDS-PAGE/Fig.2 E Unlabeled.jpg]

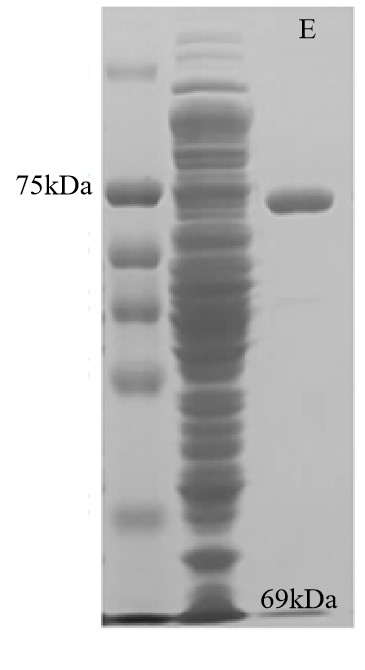

Supplement: Supplementary file 1 — Supplementary Material 1. [file 12917_2025_4980_MOESM1_ESM.zip › Uncropped_gels_blots_Figure.2/SDS-PAGE/Fig.2 E.jpg]

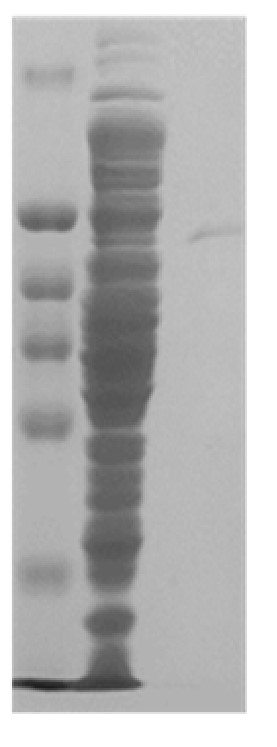

Supplement: Supplementary file 1 — Supplementary Material 1. [file 12917_2025_4980_MOESM1_ESM.zip › Uncropped_gels_blots_Figure.2/SDS-PAGE/Fig.2 F Unlabeled.jpg]

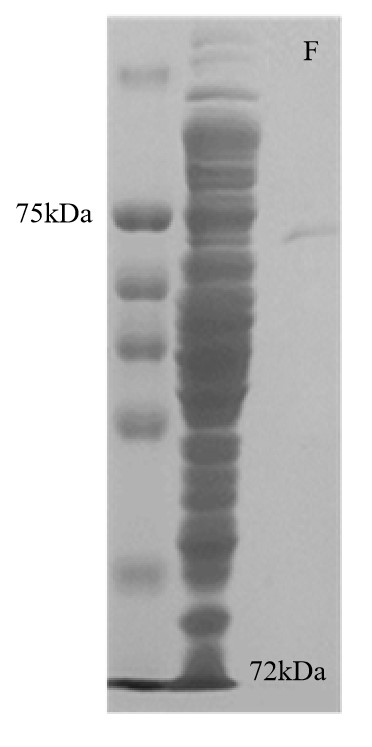

Supplement: Supplementary file 1 — Supplementary Material 1. [file 12917_2025_4980_MOESM1_ESM.zip › Uncropped_gels_blots_Figure.2/SDS-PAGE/Fig.2 F.jpg]

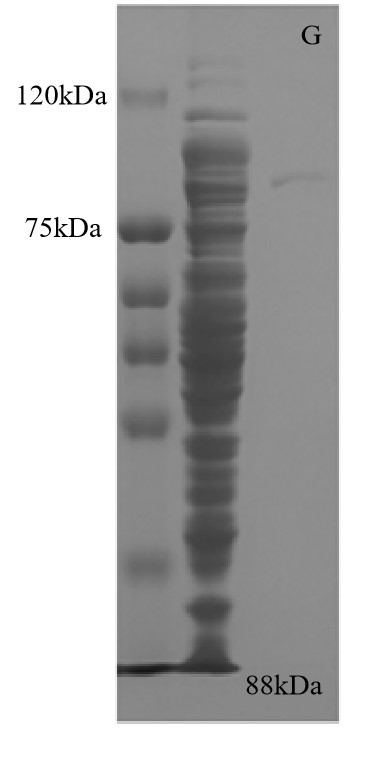

Supplement: Supplementary file 1 — Supplementary Material 1. [file 12917_2025_4980_MOESM1_ESM.zip › Uncropped_gels_blots_Figure.2/SDS-PAGE/Fig.2 G.jpg]

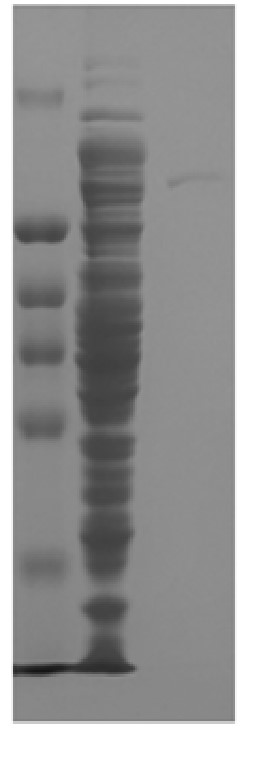

Supplement: Supplementary file 1 — Supplementary Material 1. [file 12917_2025_4980_MOESM1_ESM.zip › Uncropped_gels_blots_Figure.2/SDS-PAGE/G.jpg]

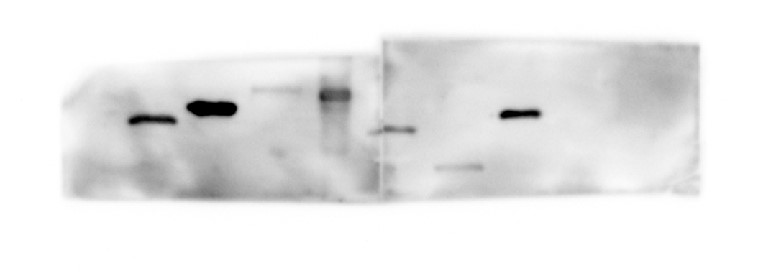

Supplement: Supplementary file 1 — Supplementary Material 1. [file 12917_2025_4980_MOESM1_ESM.zip › Uncropped_gels_blots_Figure.2/Western blot/Fig.2 A,B Unlabeled.jpg]

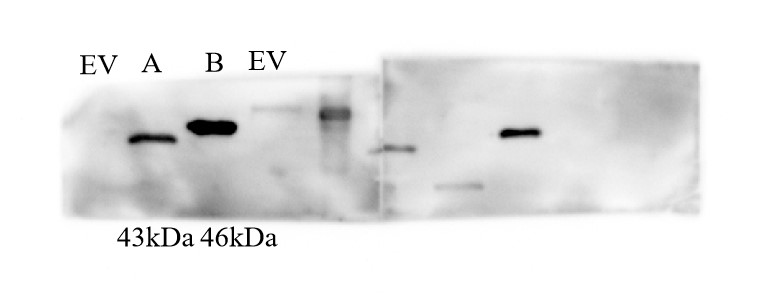

Supplement: Supplementary file 1 — Supplementary Material 1. [file 12917_2025_4980_MOESM1_ESM.zip › Uncropped_gels_blots_Figure.2/Western blot/Fig.2 A,B.jpg]

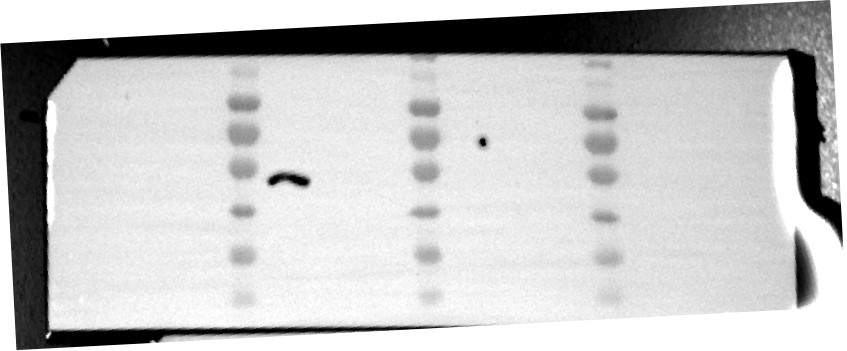

Supplement: Supplementary file 1 — Supplementary Material 1. [file 12917_2025_4980_MOESM1_ESM.zip › Uncropped_gels_blots_Figure.2/Western blot/Fig.2 C Unlabeled.jpg]

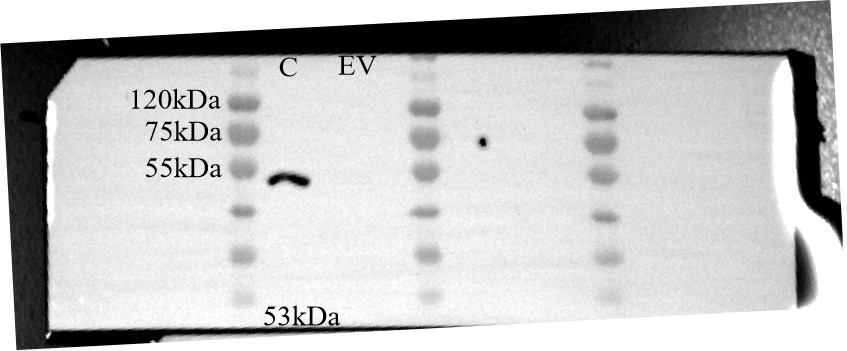

Supplement: Supplementary file 1 — Supplementary Material 1. [file 12917_2025_4980_MOESM1_ESM.zip › Uncropped_gels_blots_Figure.2/Western blot/Fig.2 C.jpg]

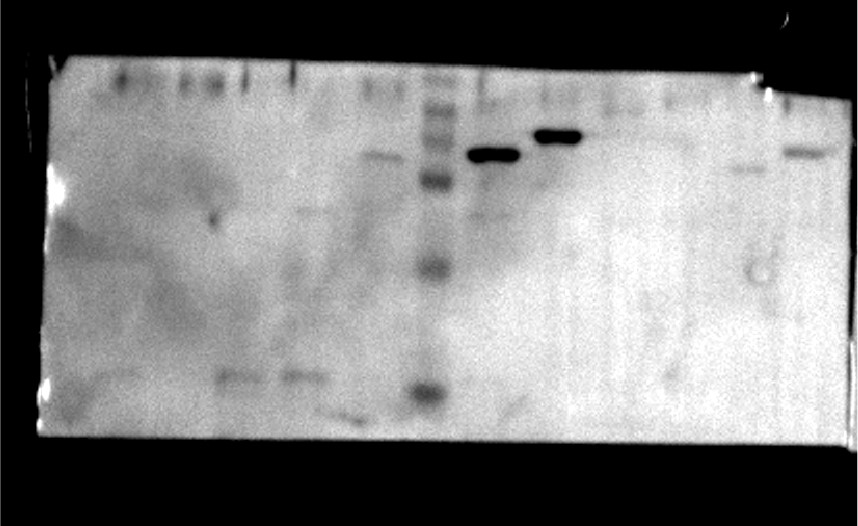

Supplement: Supplementary file 1 — Supplementary Material 1. [file 12917_2025_4980_MOESM1_ESM.zip › Uncropped_gels_blots_Figure.2/Western blot/Fig.2 D,E Unlabeled.jpg]

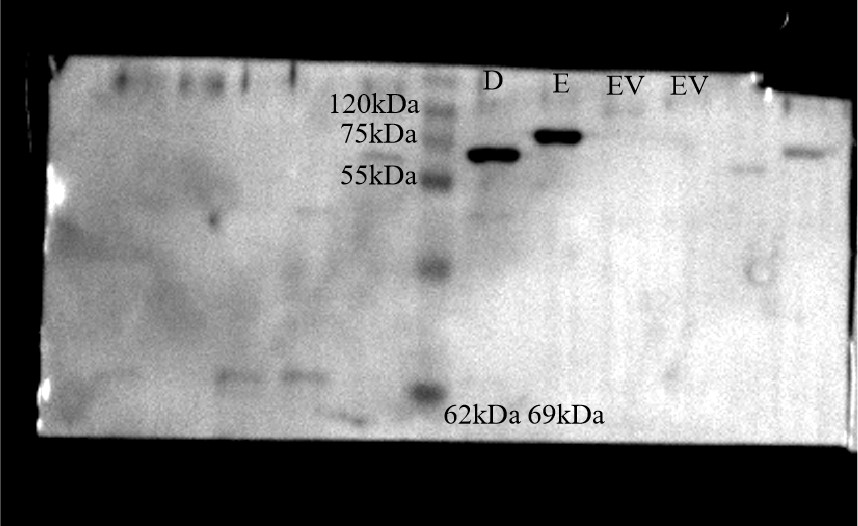

Supplement: Supplementary file 1 — Supplementary Material 1. [file 12917_2025_4980_MOESM1_ESM.zip › Uncropped_gels_blots_Figure.2/Western blot/Fig.2 D,E.jpg]

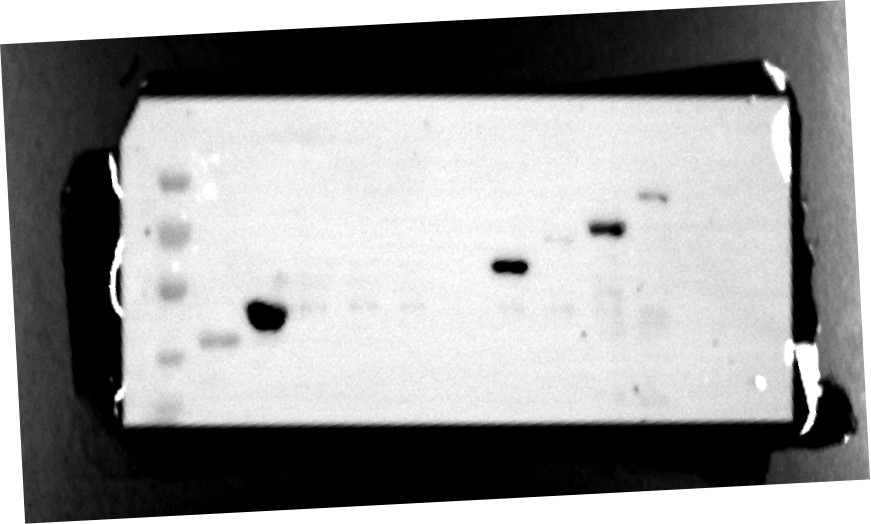

Supplement: Supplementary file 1 — Supplementary Material 1. [file 12917_2025_4980_MOESM1_ESM.zip › Uncropped_gels_blots_Figure.2/Western blot/Fig.2 F,G Unlabeled.jpg]

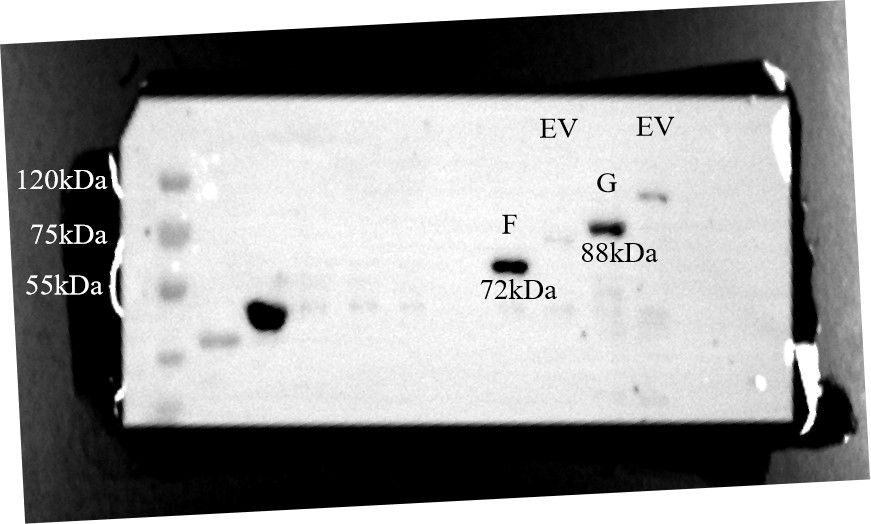

Supplement: Supplementary file 1 — Supplementary Material 1. [file 12917_2025_4980_MOESM1_ESM.zip › Uncropped_gels_blots_Figure.2/Western blot/Fig.2 F,G.jpg]
